# The isolated working guinea pig heart: A functional and electrophysiological characterisation

**DOI:** 10.1113/EP093591

**Published:** 2026-05-19

**Authors:** Grace C. Anderson‐Barker, Michael J Shattock

**Affiliations:** ^1^ School of Cardiovascular and Metabolic Medicine and Sciences, King's College London The Rayne Institute, St Thomas’ Hospital London UK

**Keywords:** contractile function, ECG, guinea pig, Isolated heart, Langendorff, working heart

## Abstract

Small animal isolated perfused hearts have been used for over 150 years for the study of cardiac physiology and pharmacology. The guinea pig heart represents the smallest mammalian heart that replicates key features of the human heart – for example, with respect to cardiac action potential duration, ion channel expression, excitation–contraction coupling and intracellular Ca^2+^ handling. In this study we describe the apparatus, instrumentation and characterisation of the isolated working guinea pig heart and utilise state‐of‐the‐art pressure–volume catheters and so forth to characterise systolic and diastolic function in this auxotonically contracting preparation. We describe novel correction formulae to allow rate‐dependent variables, such as the QT interval of the ECG and left‐ventricular pressure, to be reliably corrected for changes in heart rate.

## Abstract (highlights)

- **What is the central question of this study?**
  The aim of this study was to establish an isolated working heart model from a small animal species with a long ventricular action potential.
- **What is the main finding and its importance?**
  This work describes and characterises the isolated working guinea pig heart in sufficient detail to allow this useful preparation to be reproduced by other investigators. In addition, it describes a generic algorithm for the rate correction of electrophysiological and functional variables that are influenced by changes in heart rate (such as the QT interval or left‐ventricular pressure). Specifically, for QT interval correction, for example, this has been shown to outperform standard correction equations such as Bazett's or Fridericia's equations.

## 1 INTRODUCTION

Isolated perfused small animal hearts have been used extensively for cardiotoxicity and pharmacological testing (Bell et al., 2011; Liao et al., 2012; Louradour et al., 2023; Pouna et al., 1996; Sutherland et al., 2003; Suzer et al., 1998). While many mammalian species have been studied, hearts isolated from rats and mice appear in >70% of the isolated heart literature. Rat and mouse hearts, however, share a number of unusual physiological characteristics rendering the murine heart less than ideal as a model of human physiology. For example, murine hearts are characterised by a short action potential, elevated intracellular Na^+^, a negative force–frequency relationship and contractility that is dependent on sarcoplasmic reticulum (SR) Ca^2+^ release (Bers, 2001; Bers, Barry et al., 2003; Janssen & Periasamy, 2007; Shattock & Bers, 1989). In contrast, in guinea‐pig hearts the ventricular action potential exhibits a long plateau and excitation–contraction coupling is more similar to that seen in the human myocardium (Bers, 2001; Joukar, 2021). The guinea pig therefore represents a relatively inexpensive small animal whose heart is more similar in these regards to the human and may, therefore, provide a more suitable model for studies of intracellular Ca^2+^ handling, systolic and diastolic dysfunction and ion channel blocker/drug safety.

The majority of isolated heart studies use the Langendorff‐perfused heart – a versatile model with many applications. However, the working heart has significant advantages over the Langendorff heart when studying cardiac contractile responses. In the working heart, the left side of the heart is filled with physiological buffer that is ejected via the aorta against a defined hydrostatic ‘afterload’, which in turn determines perfusion of the coronary vasculature. This is more similar to the situation in vivo than that in the Langendorff heart where the heart contracts isovolumically against a fixed balloon, afterload is infinite and coronary perfusion is set independently.

In the isolated working heart, as in vivo, when the aortic valve is closed, contraction is initially isovolumic (although, as a result of torsion, not necessarily isometric). This is then followed, when the aortic valve opens, by a period of shortening against an afterload, and finally ventricular ejection. In this way, the isolated heart performs external work. After peak ejection, the fall in aortic pressure is initially influenced by aortic compliance and Windkessel effects. However, after the closure of the aortic valve, relaxation proceeds initially isovolumically before the mitral valve reopens and the ventricle refills. Both contraction and relaxation can therefore be considered to be auxotonic and very similar to that in vivo.

In this study we have therefore characterised the functional and electrophysiological properties of a guinea pig isolated working heart model exploiting access to pressure–volume (PV) catheters and recording systems. In addition, since many physiological variables (such as QT interval, relaxation time, etc.) vary naturally as a function of heart rate, we also demonstrate a novel method for correcting these to allow accurate assessment of electrical and contractile function independent of changes in heart rate.

## 2 METHODS

### 2.1 Ethical approval

All animal procedures were performed in compliance with Home Office guidance on the operation of the Animals (Scientific Procedures) Act 1986, the Directive 2010/63/EU of the European Parliament, and local King's College London institutional guidelines.

### 2.2 Animal housing and husbandry

All animals used in studies were male Dunkin–Hartley guinea pigs (Marshall BioResources, Huntingdon, UK) (500–700 g). Animals were group‐housed where possible, and experienced standard 12‐h light–dark cycles with free access to food and drinking water at all times. Food consisted of standard guinea pig chow, hay and dried vegetables.

### 2.3 Isolation and perfusion of guinea pig hearts

Animals were anaesthetised by intraperitoneal injection of sodium pentobarbitone (approx. 160 mg/kg) and heparin (150 I.U.). A surgical level of anaesthesia was confirmed by checking for lack of pedal reflex before removal of the heart via thoracotomy and immediate submersion in ice‐cold physiological buffer. The physiological Krebs–Henseleit buffers used for heart perfusion throughout contained (in mmol L^−1^): NaCl 114, NaHCO_3_ 24.0, KCl 4, CaCl_2_ 1.5, NaH_2_PO_4_ 1.1, MgSO_4_ 1.0, glucose 11.0, sodium pyruvate 2.0, pH 7.4 when gassed with 95% O_2_–5% CO_2_.

**FIGURE 1:**
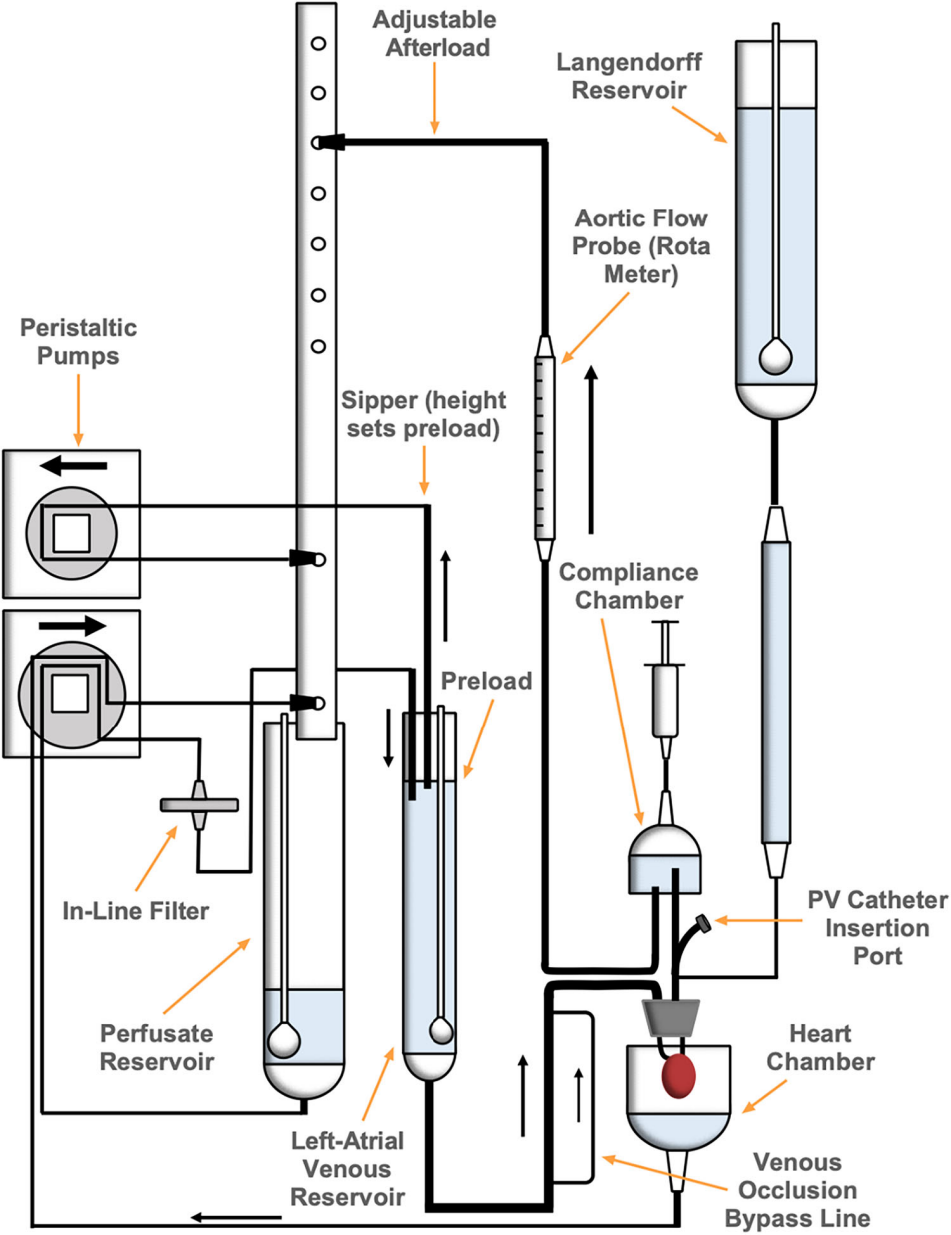
Experimental set‐up and perfusion circuit used for working guinea pig hearts. This recirculating system allows for hearts to be initially perfused in Langendorff mode while a cannula is secured in the left atrium via the opening for the pulmonary veins. Hearts are then switched to working mode whereby the left side of the heart is filled via the atrial cannula and the Langendorff reservoir is closed off. Buffer is ejected via the aorta into a Windkessel compliance chamber containing a fixed volume of air (10 mL) and from there against a fixed hydrostatic column. Aortic flow is measured by an appropriate rotameter (12–150 mL min^−1^) and afterload set by adjusting the height of the out‐flow column. Buffer is then returned to a perfusate reservoir where it is gassed and pumped via a cellulose acetate in‐line filter (porosity 5 µm) to the left atrial venous reservoir. Preload is set by adjusting the height of a sipper in this reservoir connected to a peristaltic pump set at a flow rate exceeding that of the expected cardiac output and the venous return pump. Coronary flow drips from the heart into a heart chamber and coronary flow rate can be measured with a measuring cylinder or in‐line flow meter before the perfusate is returned, via the peristaltic pump, to the perfusate reservoir. A self‐sealing pressure–volume (PV) catheter insertion port allows for the introduction of a PV catheter into the left ventricle via the aortic cannula. A venous occlusion bypass line allows partial occlusions of venous return to mimic in vivo inferior vena cava (IVC) occlusions to generate families of PV loops. The dimensions of the atrial and aortic cannulae are i.d. 2.5 mm and o.d. 3.5 mm. All tubing was silicone, minimum i.d. 2.5 mm. All glassware was water‐jacketed and maintained at 37.5 ± 0.5°C.

The ascending aorta was then exposed, and the heart immediately mounted onto a cannula before commencing flow of buffer via a gravity‐fed system. This enabled retrograde coronary perfusion at a constant pressure of 60–70 mmHg. Physiological buffer was gassed throughout (95% O_2_, 5% CO_2_) to maintain correct oxygenation and pH 7.4, and reservoir temperature was maintained using a heated bath circulator (Thermo Fisher Scientific, Waltham, MA, USA) to give a heart temperature of 37.5 ± 0.5°C. In a small number of hearts, to allow comparison with ejecting hearts, an intraventricular balloon (IVB) was inserted into the left ventricle (LV) via the pulmonary veins and mitral valve to record isovolumic contraction during Langendorff perfusion. In the majority of hearts, however, once Langendorff perfusion was established, the pulmonary vein openings in the left atria were identified, if necessary consolidated into a single opening, and cannulated. This allowed filling of the left side of the heart, and perfusion was then switched from the aortic to the pulmonary vein cannula, allowing the left side of the heart to fill and eject physiological buffer via the aortic cannula and perform external work (Figure 1). Buffer was recirculated via an oxygenation chamber and in‐line filter and the experimental set‐up allows for preload and afterload to be varied (see legend to Figure 1). Coronary flow was allowed to autoregulate at this fixed afterload.

### 2.4 Instrumentation of isolated working hearts

**FIGURE 2:**
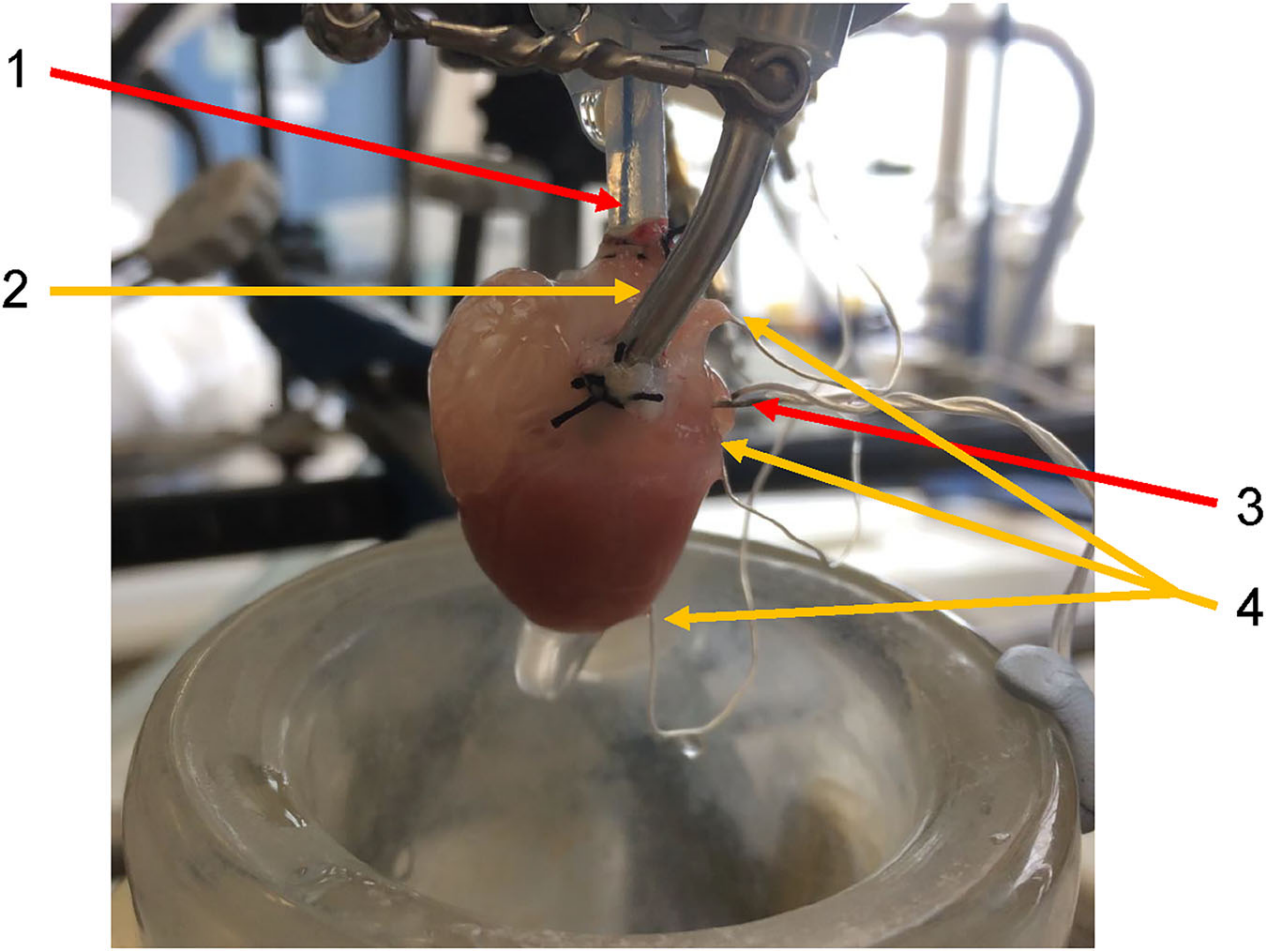
Working heart instrumentation. (1) Pressure–volume catheter within the aortic cannula. (2) Pulmonary vein cannula. (3) Bipolar pacing electrodes positioned on the right atrium. (4) ECG wires positioned on the ventricular apex (positive), right atrium (negative) and connective tissue surrounding aorta (earth).

Cardiac function was monitored via a PV catheter (Transonic Systems, Itheca, NY, USA) inserted into the LV via a self‐sealing injection port situated above the heart in the aortic outflow line. An epicardial ECG was recorded using silver wire electrodes positioned in a modified Lead II configuration. Where required, pacing was performed using bipolar silver wire electrodes positioned on the surface of the right atrium (Figure 2). Physiological data (left ventricular pressure [LVP] and ECG) were recorded using a PowerLab 8/30 and LabChart 7 software (ADInstruments, Dunedin, NZ) with the *PV Loop* and *ECG Analysis* modules.

Although this working heart set‐up allows preload and afterload to be altered (Figure 2), in the majority of these studies a preload of 15 mmHg and an afterload of 55 mmHg were used throughout as these have been as previously described to maximise contractile and metabolic efficiency (Bunger et al., 1979).

### 2.5 Investigating the effect of changing heart rate on physiological variables in the isolated working heart

Hearts were treated with low, incremental doses of ivabradine to gradually lower heart rate, before atrial pacing was used to gradually increase heart rate again in a series of small steps. Hearts were initially cannulated and allowed to stabilise in Langendorff‐mode for 15 min, before switching to working mode and allowing a further 15 min for stabilisation. Incremental concentrations of ivabradine were then added to the physiological buffer in 0.2 µM steps (up to a maximum concentration of 0.6 µM) to gradually lower heart rate to approximately 120 beats min^−1^ (bpm). Heart rate was then gradually increased again using atrial pacing (at 1.5× capture threshold) in 20 bpm steps (allowing 2 min at each pacing rate) up to a maximum heart rate of 310 bpm. This allowed ECG and functional data to be recorded across the range of physiological heart rates, and the relationship between various cardiac parameters and cardiac cycle length (i.e., RR interval) defined.

**FIGURE 3:**
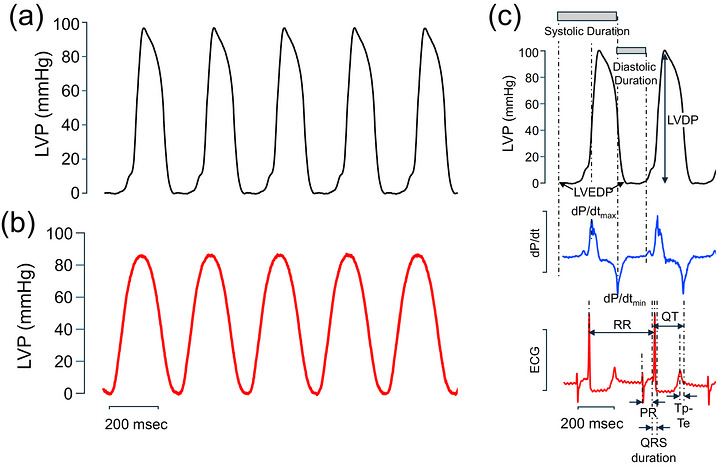
Example traces showing left ventricular pressure (LVP) measured using a pressure–volume tip‐transducer (a) and an intraventricular balloon (b) and the variables measured (c). In (a), pressure was measured using a tip‐transducer as described in an ejecting working heart and in (b), in an isovolumically contracting Langendorff preparation. Note: the symmetrical appearance and lack of true diastolic pause in the Langendorff pressure trace (b), whereas the working heart trace (a) shows a true EDP morphology and atrial ‘kick’, different rates of contraction and relaxation and an identifiable diastolic interval. Panel (c) shows example LVP, d*P*/d*t* and ECG traces and the definition of the intervals and variables measured. Cyclic variables were defined automatically in real‐time by the LabChart software, where systolic duration is defined as left ventricular end‐diastolic pressure (LVEDP) to d*P*/d*t*_min_ and diastolic duration as d*P*/d*t*_min_ to end of cycle. LVDP, left ventricular developed pressure; Tp–Te, T‐peak to T‐end interval.

For analysis, data from eight individual hearts were plotted with 10 s of data analysed for each heart rate (both paced and unpaced during ivabradine application, with data analysed after each 10 bpm reduction in heart rate). For analysis, LabChart 8 Pro with *Blood Pressure* and *ECG Analysis* modules was used. To analyse LVP variables, the *Blood Pressure Analysis* module was used to analyse LVP and new channels created for d*P*/d*t*_max_, d*P*/d*t*_min_, systolic duration, diastolic duration, time constant of relaxation (tau) and end‐diastolic pressure. ECG variables were measured using the *ECG Analysis* tool to average 10 beats, with manual correction for the detection of ECG features. Definitions of variables and intervals measured are as indicated in Figure 3c. Each variable was plotted against the corresponding RR interval or heart rate (bpm) to give the relationship between heart rate and each variable of interest, and a linear regression analysis carried out on the combined group data to give the slope and *r* ^2^ value for each parameter (see Table 1).

**TABLE 1:** Summary of correlation coefficients (*r* ^2^) for HR vs. RR for electrophysiological, systolic and diastolic variables

Group	Variable	Correlation coefficient for HR	Correlation coefficient for RR
Electrophysiology	QT	**0.793**	0.756
	Tp–Te	**0.062**	0.056
	PR	0.01996	**0.02003**
	QRS duration	**0.021**	0.010
Systolic function	LVDP	0.468	**0.489**
	d*P*/d*t*_max_	0.649	**0.691**
	Systolic duration	0.834	**0.936**
Diastolic function	LVEDP	0.380	**0.3970**
	d*P*/d*t*_min_	0.123	**0.133**
	Diastolic duration	0.926	**0.985**
	Tau	0.333	**0.365**

Correlation coefficients were taken from Figures 5, 6 and 8 and other figures (not shown). When comparing the same variable plotted vs. heart rate (HR) or RR interval (RR), the highest correlation coefficient is shown in bold. In general, electrophysiological variables were more closely correlated to HR while functional variables (systolic and diastolic) showed a closer correlation when plotted against RR Interval. LVDP, left ventricular developed pressure; LVEDP, Left ventricular end‐diastolic pressure; Tp–Te, T‐peak to T‐end interval.

### 2.6 Statistics

Where applicable, data were analysed using two‐way ANOVA with Dunnett's *post hoc* test to determine whether significant differences were present between experimental groups at each time point, and a *P‐*value of <0.05 was deemed statistically significant. Hearts were randomised to study groups, and experiments and analysis were conducted in a blinded fashion. The LabChart software used to record and identify physiological landmarks is, in general, excellent at identifying the physiological variables measured. In some PV loops in the presence of cyclopeizonic acid (CPA) the end systolic point was rounded and hard to define, and such loops were excluded from the analysis giving rise to differences in *n* numbers in some of the data sets. No data points were excluded simply because they were outliers.

## 3 RESULTS

### 3.1 Characterisation of the working guinea pig heart

Figure 3 shows a representative trace from an IVB used in a Langendorff‐perfused guinea pig heart (in red), compared to a trace obtained using a PV catheter in a working guinea pig heart (in black). From these traces there are substantial differences in the morphology of the LV pressure trace obtained with these two experimental models, with the Langendorff heart showing a very symmetrical sinusoidal shape and no appreciable diastolic interval. In comparison, the working heart shows different rates of contraction and relaxation, with an asymmetric LV pressure trace more typical of that observed in vivo. In addition, there is a greater diastolic pause with a true end‐diastolic pressure morphology. This difference in the morphology of the pressure trace and the closer approximation to what is observed in vivo gives confidence that the PV catheter provides more physiological data which may be more sensitive to changes in cardiac function. The presence of a ‘true’ end diastolic pressure also means that this parameter may provide more reliable data on diastolic function.

**FIGURE 4:**
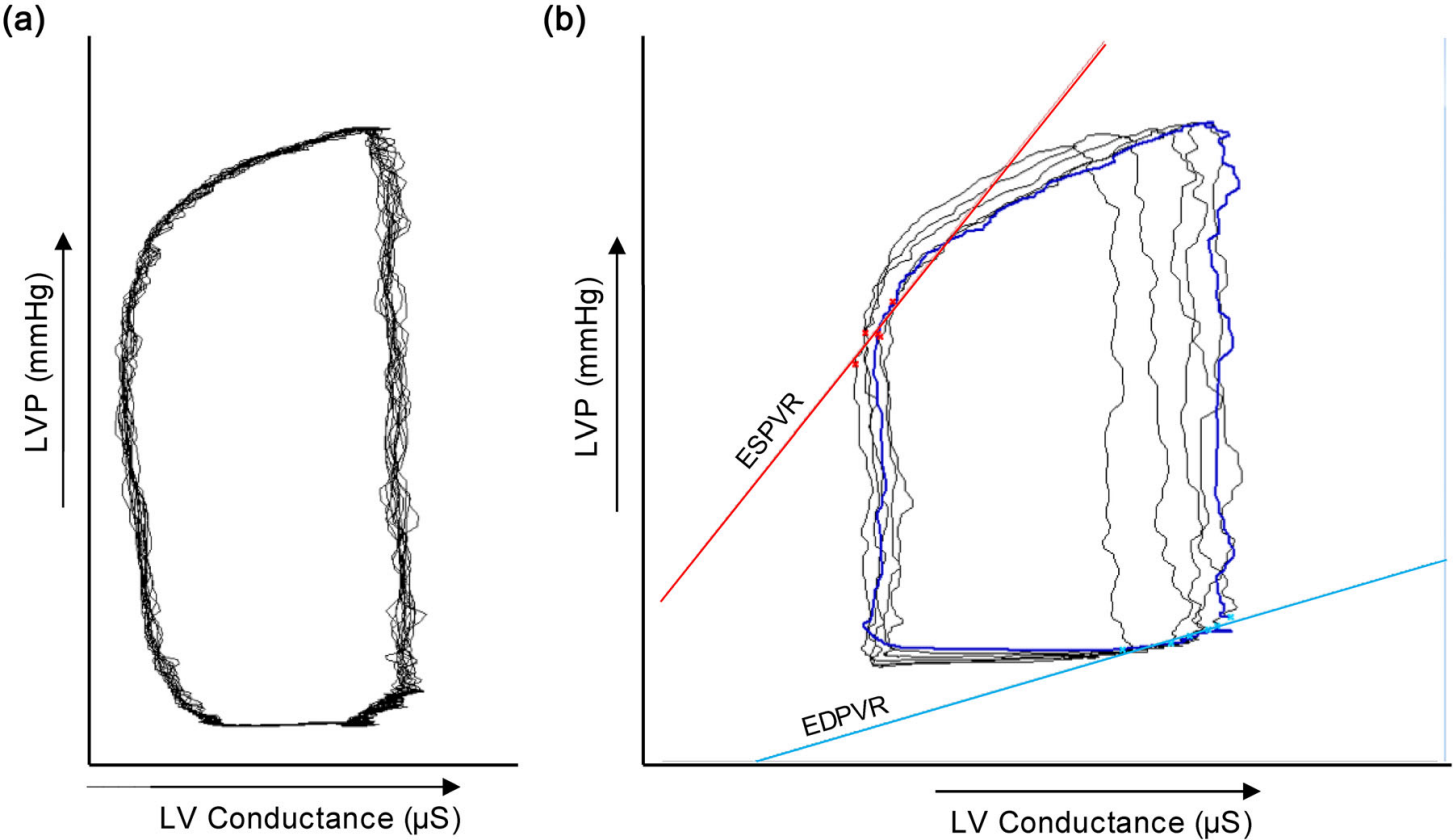
Example PV loop and ‘IVC’ occlusion from working heart set‐up. Representative examples of (a) pressure–volume (PV) loops obtained using a PV catheter within the left ventricle of an isolated working guinea pig heart and (b) modified ‘IVC’ occlusions performed by abruptly partially occluding inflow to the left side of the working heart to enable end diastolic‐ and end systolic PV relationships to be determined – referred to as preload occlusions (blue and red lines, respectively). Note: raw LV conductance data are displayed on the *x*‐axis owing to the inability to correctly calibrate volume calculation on the ADV500 PV system used. EDPVR, end‐diastolic pressure–volume relationship; ESPVR, end‐systolic pressure–volume relationship; IVC, inferior vena cava; LVP, left ventricular pressure.

Examples of the pressure–conductance loops and preload occlusions obtained in the guinea pig working heart model are shown in Figure 4. Good quality loops were obtained in most hearts, although it can be seen that the morphology of the ejection phase differs slightly from the domed appearance of loops obtained in vivo, which is likely a reflection of the artificial afterload experienced by isolated working hearts. Other phases of the cardiac cycle appeared similar to that observed in vivo, and the end diastolic point was generally well detected by LabChart PV loop analysis software when analysing occlusions; however, it was found that the rounded ejection phase often made the accurate detection of the end systolic point challenging.

Empirically, it is worth noting that in comparison to a standard Langendorff‐perfused guinea pig heart, the working heart was much more stable, showed fewer arrhythmias and maintained stable function and rhythm for many hours. This was not systematically characterised.

### 3.2 Functional and electrophysiological changes as a function of heart rate

Many physiological variables such as left ventricular developed pressure (LVDP) or QT interval are known to change as a function of heart rate. QT interval, for example, is typically corrected for changes in heart rate using a variety of equations such as Bazett's, Fridericia's, Framingham and so forth. None of these equations have been validated in the guinea pig heart and no generally applicable equations have been defined for other rate‐dependent physiological variables.

One aim of the present study was to define suitable equations for the rate correction of these variables to allow comparison across different heart rates. This is particularly useful when drugs or interventions are used experimentally that change heart rate.

**FIGURE 5:**
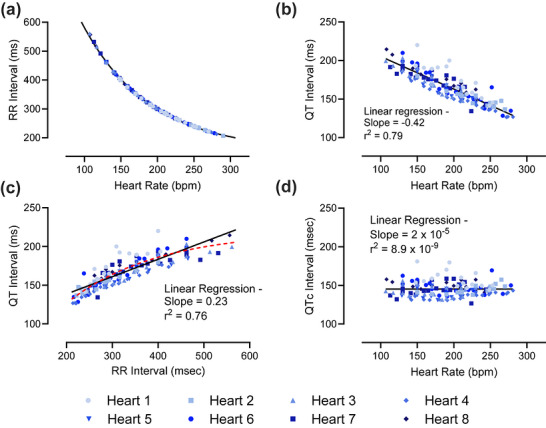
Relationships between RR interval, heart rate, QT and QTc intervals in working guinea pig hearts paced across a range of heart rates. (a) Example data showing the non‐linear relationship between heart rate and RR interval. (b) Relationship between heart rate and QT interval recorded at different pacing rates and in the presence of varying concentrations of ivabradine (0–0.6 µmol.L^−1^). (c) Data as in (b) plotted as a function of RR interval. The linear regression fit is shown in black while the red dashed line indicates the line describing a single exponential plateaux function. Note: the higher correlation coefficient (*r* ^2^) of the linear regression in (b) compared to (c) (black line) indicates QT interval data are best described as a linear function of heart rate rather than RR interval. (d) QT interval corrected for changes in heart rate (QTc) using the data in (b) and Equation 1 (see text).

**FIGURE 6:**
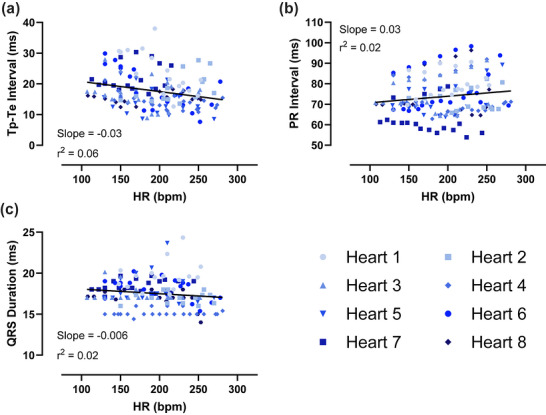
Relationship between heart rate and other ECG variables. ECG variables were measured in working guinea pig hearts paced across a range of heart rates in the presence of varying concentrations of ivabradine (0.6 µmol L^−1^). Relationships between heart rate and Tp–Te interval (a), PR interval (b), and QRS duration (c) are shown. The slope of the linear regression analysis and correlation coefficient (*r* ^2^) for each data set are shown for grouped data from all 8 hearts.

**FIGURE 7:**
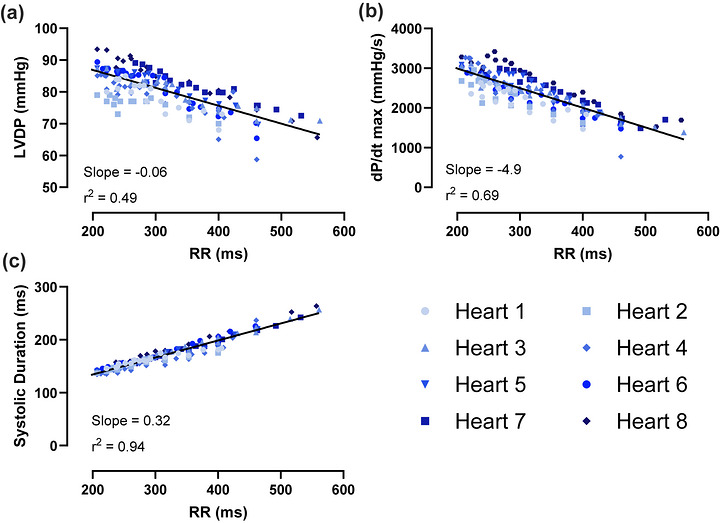
Relationship between RR interval and indices of systolic function. Systolic functional variables were measured in working guinea pig hearts paced across a range of heart rates in the presence of ivabradine (0.6 µmol L^−1^). Relationships between the RR interval and LVDP (a), d*P*/d*t*_max_ (b), and systolic duration (c) are shown. The slope of the linear regression analysis and correlation coefficient (*r* ^2^) for each data set are shown for grouped data from all 8 hearts.

**FIGURE 8:**
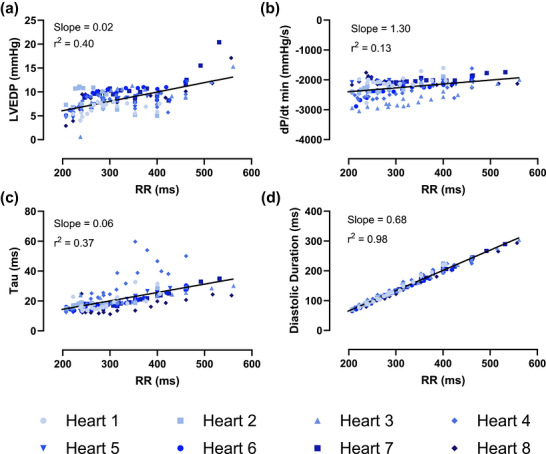
Relationship between RR interval and indices of diastolic function. Diastolic functional variables were measured in working guinea pig hearts paced across a range of heart rates in the presence of ivabradine (max. 0.6 µmol L^−1^). Relationships between RR interval and LVEDP (a), d*P*/d*t*_min_ (b), tau (c), and diastolic duration (d) are shown. The slope of the linear regression analysis and correlation coefficient (*r* ^2^) for each data set are shown for grouped data from all 8 hearts.

It is sometimes surprisingly overlooked that heart rate (in beats min^−1^, bpm) and RR interval (ms) are not linearly related (see Figure 5a). Despite this, some equations for correcting physiological variables, such as QT interval for example, assume a direct relationship between the variable and RR interval while others assume a direct relationship with heart rate. These cannot both be true and often such corrections fail at more extreme heart rates. For QT interval corrections, for example, Bazett, Framingham, Water, Fridericia and Benatar correlate QT to functions of RR interval while others (Hodges) use heart rate (for review see Haq et al., 2025). In order to determine which physiological variables were best described by linear fits to RR interval (expressed in ms) and which to heart rate (expressed as bpm), all data were plotted as a function of both (selected plots are shown in Figures 5, 6, 7, 8). For example, Figure 5 shows QT interval plotted both as a function of heart rate (Figure 5b) and of RR Interval (Figure 5c). It is clear that the QT interval is linearly related to heart rate while non‐linearly related to RR interval. Similar comparisons were made for a range of other electrophysiological and functional variables, and the correlation coefficients (*r* ^2^) derived describing ‘goodness of fit’ for these relationships are shown in Table 1. In general, electrophysiological parameters are best described as a linear function of heart rate (bpm) while function parameters are better described as a linear function of RR interval (ms).

### 3.3 Correcting electrophysiological variables for changes in heart rate

Having established that electrophysiological parameters are generally well described by a linear fit of data to heart rate (in bpm), we can use this information to derive a bespoke correction factor for heart rate in the isolated guinea pig heart. The average basal heart rate in the working guinea pig heart (HR_basal_) was determined by taking the average from eight hearts following the 30 min stabilisation period at the start of the protocol and was 242 bpm. This, combined with the slope of the relationship between heart rate and each functional parameter, allows a generic formula to be derived. This can be used to correct electrophysiological parameters (such as QT interval) for changes in heart rate.

(1) $$a\left(c\right)=a+b\left(HR_{\mathrm{basal}}-HR_{\mathrm{test}}\right)$$

where *a* is the recorded value of the electrophysiological variable of interest at any given heart rate, *b* is the slope of the relationship between that variable and HR (in bpm) (note: this can be positive or negative), HR_basal_ is the basal heart rate in this preparation (which was found to be 242 bpm), and HR_test_ is the heart rate at which the variable was recorded. This equation can be adapted for use in any preparation or species where nomograms can be generated and the value of 242 bpm is replaced with the baseline resting heart rate in that preparation. The corrected value of the variable is referred to as *a*(c) (e.g., rate‐corrected QT interval becomes QTc).

As an example, the most commonly used rate correction is for QT interval, and thus using the established basal heart rate of 242 bpm and the slope of the relationship shown in Figure 5b, Equation 1 becomes:

(2) $$\mathrm{QTc}\;=\;\mathrm{QT}+\left[-0.4231\left(242-\mathrm{HR}\right)\right]$$

The value of −0.4231 is derived from the slope of the relationship shown in Figure 5b and when these data are corrected using Equation 2, a flat line relationship between QTc and heart rate is observed showing a good correction across the range of heart rates studied (100–300 bpm) (Figure 5d). We have since demonstrated that this correction is generally applicable to human and guinea pig data and can be further refined to an adaptive formula that takes into account age‐dependent changes in the slope function of the QT–HR nomogram (Haq et al., 2025). This adaptive formula out‐performs previously used correction equations such as Bazett's, Fridericia's and so forth (Haq et al., 2025).

### 3.4 Correcting functional variables for changes in RR interval

In the same way, functional variables, which are best described by a linear fit to RR interval, a correction factor can be derived. At the average heart rate of 242 bpm, the RR interval is 248 ms. Thus, for functional variables, Equation 1 becomes:

(3) $$x\left(c\right)=x+y\left(248-\mathrm{RR}\right)$$

Where *x* is the recorded value of the functional variable of interest at any given RR interval (in ms), *y* is the slope of the relationship between that variable and RR interval (in ms) (note: this can be positive or negative), and RR is the RR interval at which the variable was recorded. The corrected value is referred to as *x*(c) (e.g., rate‐corrected LVDP becomes LVDP(c)).

### 3.5 Rate‐dependence of electrophysiological and functional variables in the working guinea pig heart

Of the ECG variables measured, as described above, the QT interval demonstrated the expected strong inverse relationship with heart rate, with faster hearts rates being associated with a progressive QT shortening (*r* ^2^ = 0.73) (Figure 5b). Other ECG variables examined, namely T‐peak to T‐end interval (Tp–Te), PR interval and QRS duration, showed only a weak correlation with heart rate (Figure 6).

The relationship between heart rate and systolic function is shown in Figure 7. LVDP demonstrated a strong negative correlation with RR interval (Figure 7a). That is, as RR interval decreases (i.e., at higher heart rates) LVDP increases, reflecting the positive force–frequency relationship in this species and in this preparation. Similarly, as RR intervals shorten (heart rate increases) d*P*/d*t*_max_ increases. This faster rate of contraction coupled with a slightly faster rate of relaxation (see Figure 8b) means the whole contractile cycle in systole is faster at higher heart rates. This results in a very clear linear relationship between RR interval and systolic duration shown in Figure 7c. Interestingly, these changes in the rate of contraction and rate of relaxation are seen in the absence of beta receptor stimulation and must therefore reflect intrinsic rate‐dependent mechanisms in excitation–contraction coupling that are independent of receptor activation. As these electrophysiological and functional variables show clear linear relationships with heart rate or RR interval, respectively, Equations 1, 2 and 3 (see above) can be used to distinguish drug‐ or protocol‐induced changes from those attributable to changes in heart rate alone.

Figure 8 shows the relationships between diastolic functional variables and RR interval. Left ventricular end‐diastolic pressure (LVEDP) (Figure 8a) and d*P*/d*t*_min_ (Figure 8b) both show a modest positive correlation with RR interval, with hearts demonstrating a higher LVEDP and slower rate of relaxation at slower heart rates. A more substantial positive tau (Figure 8c) and diastolic duration (Figure 8d) correlation with RR interval is demonstrated, indicating that hearts relax faster and hence spend a shorter time in diastole at high heart rates. Again, these variables will benefit from being presented in a corrected format using Equation 3, thus enabling results to be analysed independent of heart rate effects.

### 3.6 Influence of SERCA2 activity on electrical and contractile function

In order to determine which of these parameters provided the best indication of changes in contractile function, working guinea pig hearts were treated with incremental concentrations of cyclopeizonic acid (CPA) – the inhibitor of sarco/endoplasmic reticulum Ca^2+^‐ATPase (SERCA) function. Inhibition of SERCA should affect both systolic and diastolic function allowing various functional parameters to be assessed at each concentration of CPA. Hearts after stabilisation baseline measurements were recorded and preload occlusions performed. CPA was then added to the physiological buffer at incremental concentrations of 1, 5 and 10 µM and after 20 min, when function had stabilised, families of PV loops were generated using partial venous occlusion.

### 3.7 Measuring dysfunction in the working guinea pig heart

**FIGURE 9:**
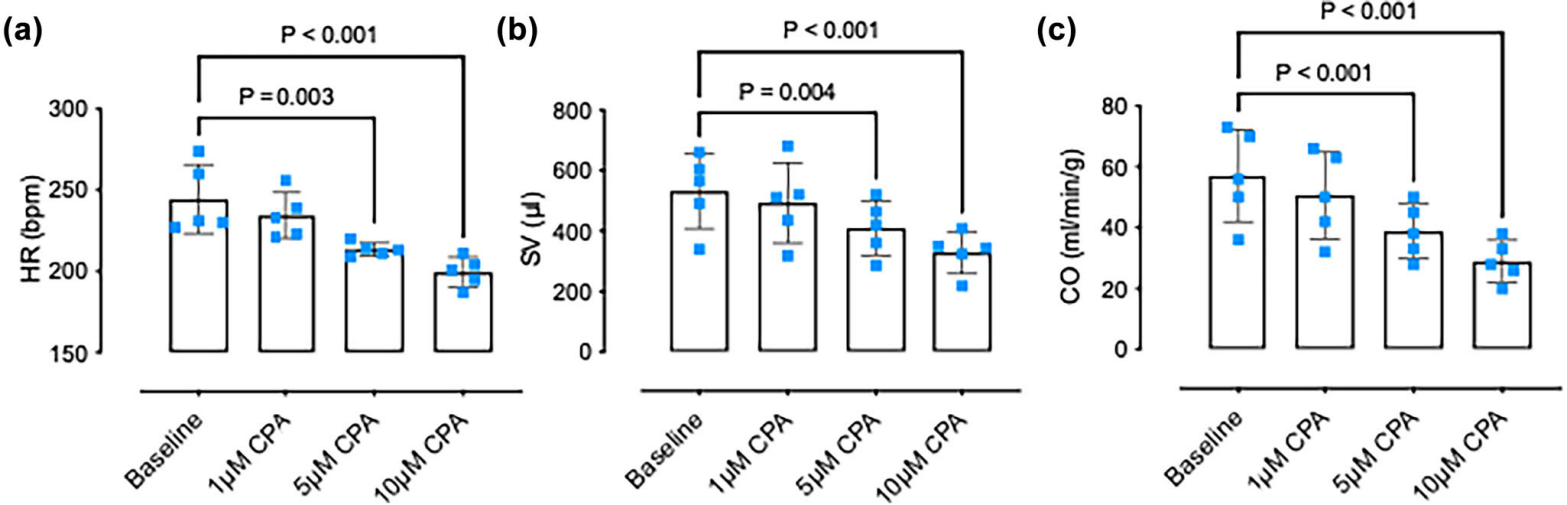
Effect of CPA on function in the working heart. Effect of incremental doses (max. 10 µmol L^−1^) of cyclopiazonic acid (CPA) on function in the isolated working guinea pig heart. The effects of each dose of CPA on heart rate (a), cardiac output (b), and stroke volume (c) are shown. Data presented as means ± SD, *n* = 5 hearts. Statistical comparisons and *P*‐values are as indicated with respect to baseline – non‐significant comparisons are not shown.

Treatment with CPA causes a reduction in heart rate, with this effect becoming significant at 5 µM (Figure 9a). This is presumed to be secondary to changes in sinoatrial (SA) node firing and a reduction in the modulation of heart rate by the SA nodal ‘calcium clock’ (Lakatta et al., 2003). This is accompanied by a reduction in cardiac output and stroke volume, both of which also show significance at 5 µM (Figures 9b,c). The fact that the reduction in cardiac output occurs alongside a reduction in stroke volume indicates that this effect is likely independent of the reduced heart rate.

**FIGURE 10:**
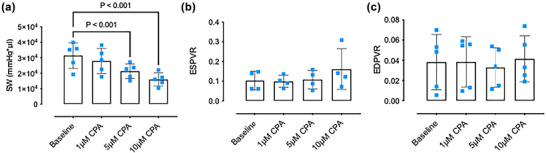
Effect of CPA on function in the working heart. Effect of incremental doses (max. 10 µmol L^−1^) of cyclopiazonic acid (CPA) on function in the isolated working guinea pig heart, as measured by pressure–volume loop parameters. The effects of each dose of CPA on stroke work (SW) (a), end systolic pressure–volume relationship (ESPVR) (b), and end diastolic pressure–volume relationship (EDPVR) (c) are shown. Data presented as means ± SD, *n* = 4–5 hearts (parameters only recorded if high‐quality pressure–volume loops were obtained). Statistical comparisons and *P*‐values are as indicated with respect to baseline – non‐significant comparisons are not shown.

Analysis of PV loops is shown in Figure 10. Stroke work (i.e., the area of the PV loop) closely mirrors the effects seen on cardiac output and stroke volume, demonstrating a significant reduction with increasing doses of CPA, reaching significance at a concentration of 5 µM (Figure 10a). The end systolic pressure–volume relationship (ESPVR) and end diastolic pressure–volume relationship (EDPVR) were also examined but showed no change at any dose of CPA (Figure 10b,c). The EDPVR in particular demonstrates large variation between hearts and thus is unlikely to detect changes in cardiac function with the group sizes used in these studies.

**FIGURE 11:**
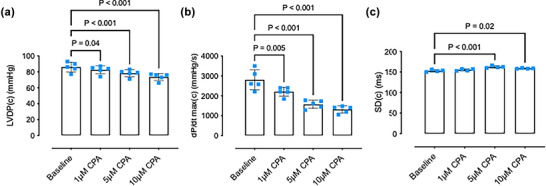
Effect of CPA on systolic function in the working heart. Effect of incremental doses (max. 10 µmol L^−1^) of cyclopiazonic acid (CPA) on systolic function in the isolated working guinea pig heart. The effects of each dose of CPA on rate‐corrected LVDP (a), d*P*/d*t*_max_ (b), and systolic duration (SD) (c). Data presented as means ± SD, *n* = 4–5 hearts. Statistical comparisons and *P*‐values are as indicated with respect to baseline – non‐significant comparisons are not shown.

Treatment with CPA enabled a clear impairment of systolic function to be detected in working guinea pig hearts (Figures 9 and 10); however, the change in heart rate will also influence contractility. To account for this we have used the correction factors described in Equation 3 (above) to correct LVDP, d*P*/d*t*_max_ and systolic duration to examine rate‐independent changes induced by CPA. Inhibiting SR Ca^2+^ release with CPA dose‐dependently reduced LVDP, slowed d*P*/d*t*_max_ and prolonged systolic duration – effects which are independent of heart rate (Figure 11).

**FIGURE 12:**
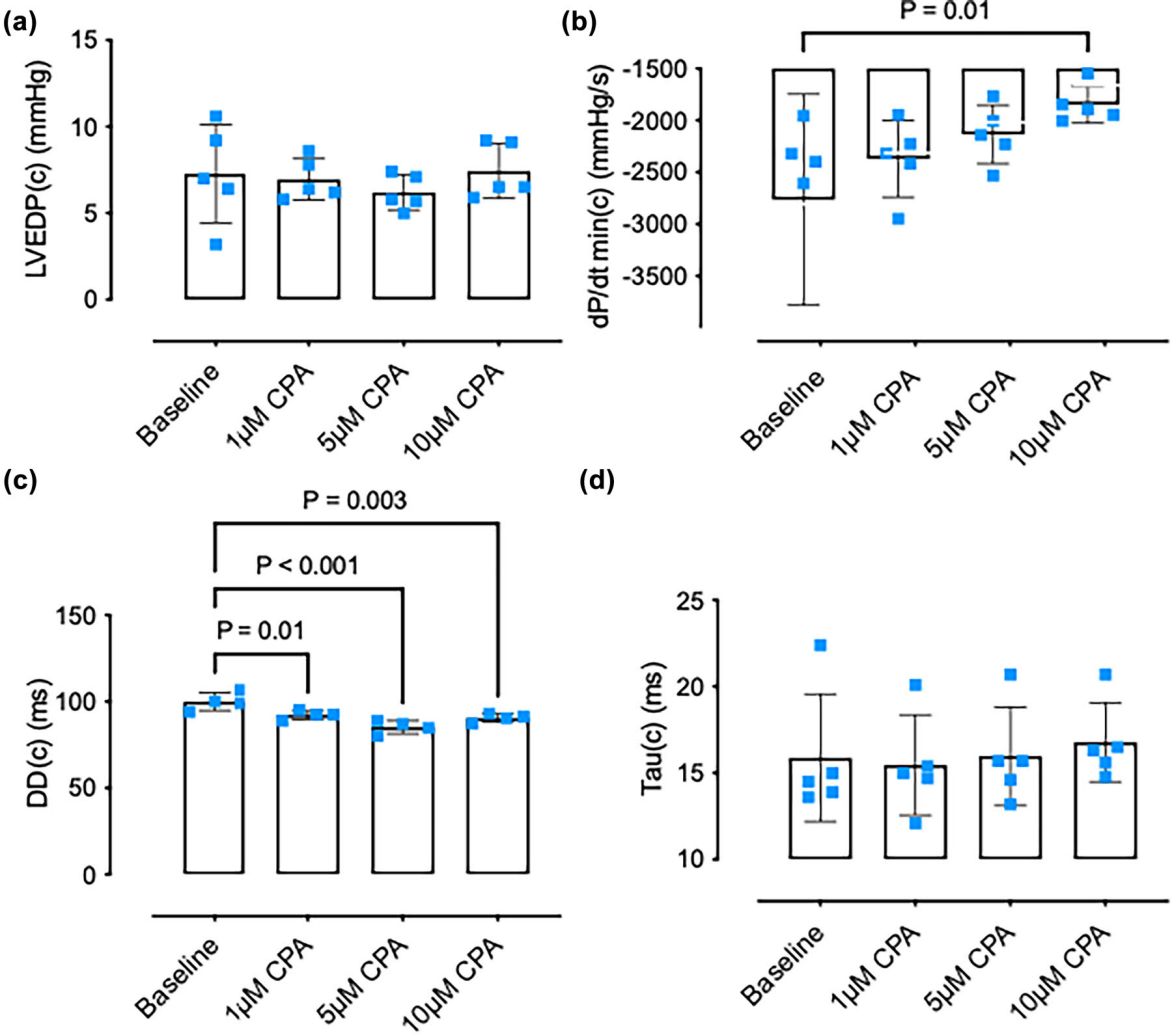
Effect of CPA on diastolic function in the working heart. Effect of incremental doses (max. 10 µmol L^−1^) of cyclopiazonic acid (CPA) on diastolic function in the isolated working guinea pig heart. The effects of each dose of CPA on rate‐corrected LVEDP (a), d*P*/d*t*_min_ (b), diastolic duration (DD) (c), and tau (d). Data presented as means ± SD, *n* = 4–5 hearts. Statistical comparisons and *P*‐values are as indicated with respect to baseline – non‐significant comparisons are not shown.

Diastolic function was also impaired in hearts treated with CPA (Figure 12). Interestingly, rate‐corrected LVEDP(c) was insensitive to CPA inhibition. The rate of pressure decline in the ventricle did respond to CPA treatment, but with d*P*/d*t*_min_(c) showing a dose‐dependent reduction in response to CPA, although this did not reach significance until 10 µM. Associated with the prolongation in rate‐corrected systolic duration (Figure 11c), CPA reciprocally and dose‐dependently reduced rate‐corrected diastolic duration.

## 4 DISCUSSION

In this study we describe a guinea pig isolated working heart model, which has significant advantages over other small animal isolated hearts in terms of both functional and electrophysiological relevance to the human heart. This model allows the recording of physiologically relevant functional variables during auxotonic cardiac contraction including cardiac output, stroke volume, cardiac work, high‐fidelity d*P*/d*t* and the generation of pressure–volume loops from which indices of systolic (ESPVR) and diastolic (EDPVR) function can be derived. In addition, the guinea pig heart represents a cost‐efficient small animal heart model with cardiac electrophysiology similar to that in the human heart.

Functional and electrophysiological variables often vary with heart rate. In 1871 Henry Bowditch originally described the positive ‘staircase’ relating heart rate to the force of contraction in a frog heart (Bowditch, 1871). Since then it has been recognised that this is both preparation and species‐dependent such that measurements of contractility need to take into account any underlying change in heart rate.

Similarly, the relationship between heart rate and electrophysiological variables such as action potential duration and its surrogate the QT interval of the ECG are rate‐dependent. The long cardiac action potential which underlies the QT interval was first recorded and recognised by Burdon‐Sanderson and Page in 1884, and in 1920 Bazett provided the first mathematical description of the rate‐dependence of the QT interval (Bazett, 1920; Burdon‐Sanderson & Page, 1884). These rate‐dependent effects can be avoided by pacing the preparation at a constant rate, but this is not always feasible (e.g., in vivo) or desirable (when heart rate is a variable also under investigation). In this study we describe and validate equations to allow functional and electrophysiological variables measured in the isolated perfused guinea pig heart to be corrected for heart rate to allow drug or intervention‐induced changes to be distinguished from those induced by rate changes alone. This approach, with suitably plotted nomograms and these equations, is generally applicable to other rate‐dependent processes recorded in vivo or in other preparations. Using this method we have developed an adaptive correction equation for correcting the QT interval for changes in heart rate in guinea pig and human ECGs that out‐performs established equations such as Bazett's, Fridericia's and so forth – see Supporting information Appendix and Haq et al. (2025). It should be noted that the specific numbers quoted in Equation 2 for the slope of the QT–heart rate relationship (Equation 1: *b*) and basal heart rate (Equation 1: HR_basal_) are only applicable to hearts isolated from male guinea pigs aged 6–9 weeks. A more flexible ‘adaptive’ approach, taking into account age‐dependent changes in QT and basal heart rate, is described in Haq et al. (2025). Researchers would be encouraged to use the basic relationship described in Equation 1 but to derive their own bespoke variables ‘*b*’ and HR_basal_ for their own cohorts and conditions. Female guinea pigs were not studied here and these numbers may also be expected to be subject to sex‐dependent differences.

In the present study we have used uncalibrated volume measurements made using an admittance catheter. These recordings were uncalibrated as the volume calculation relies on the recording system being calibrated for the conductance (resistivity) of the blood within the LV (Clark & Marber, 2013). The ADV500 PV System used in this study does not include a calibration range wide enough to accommodate the conductance of the crystalloid Krebs buffers used. This can be achieved by permanently altering an internal calibration but, as this machine was shared across in vivo and in vitro experimenters, this was not done. In our studies it was possible to measure stroke volume from measurements of cardiac output and heart rate, but the inability to accurately calibrate LV volume means that in our system it was not possible to measure ejection fraction (EF). We would therefore recommend using a dedicated device with a wider resistivity range or one in which this internal calibration can be permanently altered to allow calibrated volumes and real‐time estimates of EF.

The isolated guinea pig heart is particularly useful for studies of cardiotoxity and drug safety screening (Guo et al., 2009; Xu et al., 2022). Drug‐induced torsade de pointes (TdP) is a major concern in preclinical drug development. While not without its limitations (Hamlin et al., 2004; O'Hara & Rudy, 2012), the isolated perfused guinea pig heart is a useful medium‐throughput cost‐effective small animal heart model that recapitulates many of the features of the human heart (Guo et al., 2009). The guinea pig is the smallest commonly used laboratory mammal with a prolonged cardiac action potential whose prolongation depends strongly on the rapid and slow delayed rectifier K^+^ currents (*I*_Kr_ and *I*_Ks_), the same currents that dominate human ventricular repolarization and are frequent drug targets (Joukar, 2021; Sanguinetti & Jurkiewicz, 1991). In addition, rate‐dependent drug responses mirror human cardiomyocyte behaviour. For example, dofetilide, a selective *I*_Kr_ blocker, produces frequency‐dependent action potential prolongation in isolated guinea‐pig ventricular myocytes recapitulating human QT dynamics and hERG/*I*_Kr_ pharmacology (Jurkiewicz & Sanguinetti, 1993).

Critical for the use of isolated hearts for drug screening and TdP liability is the ability to measure QT prolongation and correct this accurately for drug‐induced changes in heart rate. The algorithm described in this study allows for accurate detection of rate‐corrected QTc prolongation. The morphology of the T wave is also used as an index of arrhythmogenicity and in particular Tp–Te is assumed to be a measure of ventricular dispersion of repolarisation (Antzelevitch, 2001; Zhu et al., 2009). In the present study, Tp–Te was quite variable and showed only a very modest dependence of heart rate – with higher heart rates associated with slightly shorter Tp–Te (i.e., decreased dispersion). The algorithm described in Equation 1 could again be used to correct changes in Tp–Te for these small changes induced by rate.

The next question that was addressed when characterising the working guinea pig heart was how contractile dysfunction could be measured, that is, which functional variables would provide sensitive and reliable detection of changes to systolic and diastolic function. For example, impaired calcium reuptake into the sarcoplasmic reticulum is a defining feature of heart failure that contributes to diastolic and systolic dysfunction (Bers, Eisner et al., 2003; Denniss et al., 2020; Kranias & Hajjar, 2012). We have therefore treated healthy hearts with incremental doses of CPA to impair SERCA activity (Pery‐Man et al., 1993; Schwinger et al., 1997; Takahashi et al., 1995).

The results demonstrate that the effects of CPA are easily apparent from basic cardiac parameters such as heart rate, cardiac output and stroke volume. However, when looking at data from ‘pressure–volume’ loops, changes to stroke work precisely mirrored those seen in cardiac output and stroke volume and therefore did not provide further useful information. When looking at the ESPVR and EDPVR obtained from preload occlusions, no change in systolic and diastolic function was apparent using this approach. Indeed, the ESPVR did not successfully detect systolic impairment at any concentration of CPA, and it was found that the LabChart PV loop analysis module was not able to reliably identify the true end systolic point, given the more sloping appearance of the ejection phase from loops obtained in the working heart. Similarly, the reported EDPVR using this approach was found to be so variable as to be unable to detect any change in diastolic function. Therefore, these loop parameters were not considered to be suitable for assessing changes to ventricular function. Parameters collected from the LV pressure trace, on the other hand, were found to be dose‐dependently affected by CPA treatment.

The unique analysis described here for correcting LVDP, d*P*/d*t*_max_ and systolic duration for changes in heart rate enables comparison uncomplicated by this confounder. d*P*/d*t*_max_ was the most sensitive variable, showing a significant reduction from the lowest concentration of CPA. The results for diastolic function appeared to be a little more complicated, with LVEDP(c) found to be somewhat variable and unable to detect changes to diastolic function. Corrected d*P*/d*t*_min_ demonstrated concentration‐dependent effects of CPA indicating a slowing of maximum rate of relaxation due to reduced SR calcium re‐uptake. Systolic duration was prolonged reflecting a combination of a slowed rate of contraction due to reduced SR Ca^2+^ load and a slowing in the early phase of relaxation.

This study describes in detail the apparatus, instrumentation and procedures necessary for functional and electrophysiological measurements to be made in an isolated working guinea pig heart. This allows pharmacological, physiological and toxicological studies in a relatively low‐cost in vitro small animal heart where a long cardiac action potential, more human‐like electrophysiology and calcium handling are desirable. Importantly we also describe an improved method for correction of rate‐dependent physiological variables, such as the QT interval of the ECG. In this example, this rate corrected QTc has been shown to out‐perform established corrections such as Bazett's, Federicia's and so forth (Haq et al., 2025).

## AUTHOR CONTRIBUTIONS

Michael J. Shattock: funding acquisition, conceptualisation, methodology, project administration, supervision, formal analysis, writing – original draft. Grace C. Anderson‐Barker: conceptualisation, methodology, investigation, formal analysis, writing – review and editing. Both authors have read and approved the final version of this manuscript and agree to be accountable for all aspects of the work in ensuring that questions related to the accuracy or integrity of any part of the work are appropriately investigated and resolved. All persons designated as authors qualify for authorship, and all those who qualify for authorship are listed.

## CONFLICT OF INTEREST

The authors declare they have no conflicts of interest.

## Supporting information



Appendix: The isolated working guinea pig heart: A functional and electrophysiological characterisation.

## Data Availability

The LabChart files (ADInstruments, Australia) containing the original digitised recordings and the analysis and graph‐plotting files containing the numerical data, graphs, curve‐fitting and statistical analysis, are deposited in FigShare (10.6084/m9.figshare.31820152).

## References

[eph70313-bib-0001] Antzelevitch, C. (2001). Tpeak‐Tend interval as an index of transmural dispersion of repolarization. European Journal of Clinical Investigation, 31(7), 555–557. 11454006 10.1046/j.1365-2362.2001.00849.x

[eph70313-bib-0002] Bazett, H. C. (1920). An analysis of the time‑relations of electrocardiograms. Heart, 7(2), 353–370.

[eph70313-bib-0003] Bell, R. M. , Mocanu, M. M. , & Yellon, D. M. (2011). Retrograde heart perfusion: The Langendorff technique of isolated heart perfusion. Journal of Molecular and Cellular Cardiology, 50(6), 940–950. 21385587 10.1016/j.yjmcc.2011.02.018

[eph70313-bib-0004] Bers, D. M. (2001). Excitation‐contraction coupling and cardiac contractile force. Springer.

[eph70313-bib-0005] Bers, D. M. , Barry, W. H. , & Despa, S. (2003). Intracellular Na^+^ regulation in cardiac myocytes. Cardiovascular Research, 57(4), 897–912. 12650868 10.1016/s0008-6363(02)00656-9

[eph70313-bib-0006] Bers, D. M. , Eisner, D. A. , & Valdivia, H. H. (2003). Sarcoplasmic reticulum Ca^2+^ and heart failure: Roles of diastolic leak and Ca^2+^ transport. Circulation Research, 93(6), 487–490. 14500331 10.1161/01.RES.0000091871.54907.6B

[eph70313-bib-0007] Bowditch, H. P. (1871). Über die Eigenthümlichkeiten der Reizbarkeit, welche die Muskelfasern des Herzens zeigen. Berichte über die Verhandlungen der Königlich‐Sächsischen Gesellschaft der Wissenschaften, Mathematisch‐Physische Klasse, 23, 652–689.

[eph70313-bib-0008] Bunger, R. , Sommer, O. , Walter, G. , Stiegler, H. , & Gerlach, E. (1979). Functional and metabolic features of an isolated perfused guinea pig heart performing pressure‐volume work. Pflügers Archiv European Journal of Physiology, 380(3), 259–266. 573465 10.1007/BF00582904

[eph70313-bib-0009] Burdon‐Sanderson, J. , & Page, F. J. M (1884). On the electrical phenomena of the excitatory process in the heart of the frog and of the tortoise, as investigated photographically. The Journal of Physiology, 4(6), 327–386. 10.1113/jphysiol.1884.sp000134 PMC1484986 16991337

[eph70313-bib-0010] Clark, J. E. , & Marber, M. S. (2013). Advancements in pressure‐volume catheter technology—stress remodelling after infarction. Experimental Physiology, 98(3), 614–621. 23064506 10.1113/expphysiol.2012.064733

[eph70313-bib-0011] Denniss, A. L. , Dashwood, A. M. , Molenaar, P. , & Beard, N. A. (2020). Sarcoplasmic reticulum calcium mishandling: Central tenet in heart failure? Biophysical Reviews, 12(4), 865–878. 32696300 10.1007/s12551-020-00736-y PMC7429633

[eph70313-bib-0012] Guo, L. , Dong, Z. &. , & Guthrie, H. (2009). Validation of a guinea pig Langendorff heart model for assessing potential cardiovascular liability of drug candidates. Journal of Pharmacological and Toxicological Methods, 60(2), 130–151. 19616638 10.1016/j.vascn.2009.07.002

[eph70313-bib-0013] Hamlin, R. L. , Cruze, C. A. , Mittelstadt, S. W. , Kijtawornrat, A. , Keene, B. W. , Roche, B. M. , Nakayama, T. , Nakayama, H. , Hamlin, D. M. &. , & Arnold, T. (2004). Sensitivity and specificity of isolated perfused guinea pig heart to test for drug‐induced lengthening of QTc. Journal of Pharmacological and Toxicological Methods, 49(1), 15–23. 14670690 10.1016/j.vascn.2003.08.003

[eph70313-bib-0014] Haq, K. T. , McLean, K. M. , Anderson‐Barker, G. C. , Berul, C. I. , Shattock, M. J. , & Posnack, N. G. (2025). Validation of a demography‐based adaptive QT correction formula using pediatric and adult datasets acquired from humans and guinea pigs. Circulation: Arrhythmia and Electrophysiology, 18(2), e013237. 39895520 10.1161/CIRCEP.124.013237 PMC12884567

[eph70313-bib-0015] Janssen, P. M. , & Periasamy, M. (2007). Determinants of frequency‐dependent contraction and relaxation of mammalian myocardium. Journal of Molecular and Cellular Cardiology, 43(5), 523–531. 17919652 10.1016/j.yjmcc.2007.08.012 PMC2093987

[eph70313-bib-0016] Joukar, S. (2021). A comparative review on heart ion channels, action potentials and electrocardiogram in rodents and human: Extrapolation of experimental insights to clinic. Laboratory Animal Research, 37(1), 25. 34496976 10.1186/s42826-021-00102-3 PMC8424989

[eph70313-bib-0017] Jurkiewicz, N. K. , & Sanguinetti, M. C. (1993). Rate‐dependent prolongation of cardiac action potentials by a methanesulfonanilide class III antiarrhythmic agent. Specific block of rapidly activating delayed rectifier K^+^ current by dofetilide. Circulation Research, 72(1), 75–83. 8417848 10.1161/01.res.72.1.75

[eph70313-bib-0018] Kranias, E. G. , & Hajjar, R. J. (2012). Modulation of cardiac contractility by the phospholamban/SERCA2a regulatome. Circulation Research, 110(12), 1646–1660. 22679139 10.1161/CIRCRESAHA.111.259754 PMC3392125

[eph70313-bib-0019] Lakatta, E. G. , Maltsev, V. A. , Bogdanov, K. Y. , Stern, M. D. , & Vinogradova, T. M. (2003). Cyclic variation of intracellular calcium: A critical factor for cardiac pacemaker cell dominance. Circulation Research, 92(3), e45–e50. 12595348 10.1161/01.res.0000055920.64384.fb

[eph70313-bib-0020] Liao, R. , Podesser, B. K. , & Lim, C. C. (2012). The continuing evolution of the Langendorff and ejecting murine heart: New advances in cardiac phenotyping. American Journal of Physiology. Heart and Circulatory Physiology, 303(2), H156–H167. 22636675 10.1152/ajpheart.00333.2012 PMC3404701

[eph70313-bib-0021] Louradour, J. , Ottersberg, R. , Segiser, A. , Olejnik, A. , Martínez‐Salazar, B. , Siegrist, M. , Egle, M. , Barbieri, M. , Nimani, S. , Alerni, N. , Döring, Y. , Odening, K. E. , & Longnus, S. (2023). Simultaneous assessment of mechanical and electrical function in Langendorff‐perfused ex‐vivo mouse hearts. Frontiers in Cardiovascular Medicine, 10, 1293032. 38028448 10.3389/fcvm.2023.1293032 PMC10663365

[eph70313-bib-0022] O'Hara, T. , & Rudy, Y. (2012). Quantitative comparison of cardiac ventricular myocyte electrophysiology and response to drugs in human and nonhuman species. American Journal of Physiology–Heart and Circulatory Physiology, 302(5), H1023–H1030. 22159993 10.1152/ajpheart.00785.2011 PMC3311457

[eph70313-bib-0023] Pery‐Man, N. , Chemla, D. , Coirault, C. , Suard, I. , Riou, B. , & Lecarpentier, Y. (1993). A comparison of cyclopiazonic acid and ryanodine effects on cardiac muscle relaxation. American Journal of Physiology, 265(4), H1364–H1372. 8238424 10.1152/ajpheart.1993.265.4.H1364

[eph70313-bib-0024] Pouna, P. , Bonoron‐Adèle, S. , Gouverneur, G. , Tariosse, L. , Besse, P. , & Robert, J. (1996). Development of the model of rat isolated perfused heart for the evaluation of anthracycline cardiotoxicity and its circumvention. British Journal of Pharmacology, 117(7), 1593–1599. 8730759 10.1111/j.1476-5381.1996.tb15326.x PMC1909465

[eph70313-bib-0025] Sanguinetti, M. C. , & Jurkiewicz, N. K. (1991). Delayed rectifier outward K^+^ current is composed of two currents in guinea pig atrial cells. American Journal of Physiology, 260(2), H393–H399. 1899980 10.1152/ajpheart.1991.260.2.H393

[eph70313-bib-0026] Schwinger, R. H. G. , Brixius, K. , Bavendiek, U. , Hoischen, S. , Müller‐Ehmsen, J. , Bölck, B. , & Erdmann, E. (1997). Effect of cyclopiazonic acid on the force‐frequency relationship in human nonfailing myocardium. The Journal of Pharmacology and Experimental Therapeutics, 283(1), 286–292. 9336335

[eph70313-bib-0027] Shattock, M. J. , & Bers, D. M. (1989). Rat vs. rabbit ventricle: Ca flux and intracellular Na assessed by ion‐selective microelectrodes. American Journal of Physiology. Cell Physiology, 256(4), C813–C822. 10.1152/ajpcell.1989.256.4.C813 2705515

[eph70313-bib-0028] Sutherland, F. J. , Shattock, M. J. , Baker, K. E. , & Hearse, D. J. (2003). Mouse isolated perfused heart: Characteristics and cautions. Clinical and Experimental Pharmacology and Physiology, 30(11), 867–878. 14678252 10.1046/j.1440-1681.2003.03925.x

[eph70313-bib-0029] Suzer, O. , Suzer, A. , Aykac, Z. , & Ozuner, Z. (1998). Direct cardiac effects in isolated perfused rat hearts measured at increasing concentrations of morphine, alfentanil, fentanyl, ketamine, etomidate, thiopentone, midazolam and propofol. European Journal of Anaesthesiology, 15(4), 480–485. 9699107

[eph70313-bib-0030] Takahashi, S. , Kato, Y. , Adachi, M. , Agata, N. , Tanaka, H. , & Shigenobu, K. (1995). Effects of cyclopiazonic acid on rat myocardium: Inhibition of calcium uptake into sarcoplasmic reticulum. The Journal of Pharmacology and Experimental Therapeutics, 272(3), 1095–1100. 7891321

[eph70313-bib-0031] Xu, X. , Yin, Y. , Li, D. , Yao, B. , Zhao, L. , Wang, H. , Wang, H. , Dong, J. , Zhang, J. , & Peng, R. (2022). Vicious LQT induced by a combination of factors different from hERG inhibition. Frontiers in Pharmacology, 13, 930831. 35935820 10.3389/fphar.2022.930831 PMC9354841

[eph70313-bib-0032] Zhu, T. G. , Patel, C. , Martin, S. , Quan, X. , Wu, Y. , Burke, J. F. , Chernick, M. , Kowey, P. R. , & Yan, G. X. (2009). Ventricular transmural repolarization sequence: Its relationship with ventricular relaxation and role in ventricular diastolic function. European Heart Journal, 30(3), 372–380. 19147608 10.1093/eurheartj/ehn585

