# Supplementary material for: The isolated working guinea pig heart: A functional and electrophysiological characterisation
Source: Exp Physiol. 2026 May 19;111(7):3137–51. doi: 10.1113/EP093591 (PMC13327299; doi:10.1113/EP093591)
Supplement: Supplementary file 1 — Appendix: The isolated working guinea pig heart: A functional and electrophysiological characterisation. [file EPH-111-3137-s001.docx]

**Appendix:**

***The Isolated Working Guinea Pig Heart:***

*A functional and electrophysiological characterisation.*

Grace C Anderson-Barker and Michael J Shattock

**Example ECGs recorded at two different pacing rates**

***Figure S1:*** Example ECGs and LV pressure traces recorded paced at two different heart rates in the guinea pig isolated working heart.

**Comparing equations frequently used for QTc correction**

In this study we have used bespoke correction formulae to correct rate-dependent physiological variables for changes in heart rate in adult male guinea pig hearts. The most common type of correction used in this context is the correction of the QT interval of the ECG to generate a corrected QT (QTc). Many previous studies have unsurprisingly concluded that bespoke corrections for a specific population cohort give the most accurate results. Numerous studies have investigated this relationship and shown it to be both age and sex specific. While the generic equations described here are applicable to other species, ages and sexes, the specific slopes and resting heart rates are unique to adult male guinea pig hearts under these isolated heart conditions.

The most frequently used QTc correction equations are Bazett’s and Fridericia’s. These are not only known to be unreliable at high and low heart rates but have often also been used in the literature to correct QT intervals recorded in species and age-groups for which they were never intended.

Some QTc correction formulae assume the QT interval of the ECG has a linear relationship with heart rate while others assume it is linear with the RR interval. As is shown in Figure 3, both cannot be true. To account for this some formulae use non-linear functions. In this supplement we have taken the same data set from our guinea pig working hearts (shown in Figure 3) and compared the performance of the Anderson-Barker equation (described here) to that of the following commonly used QTc correction equations:

| **Equation name** | **Formula** | **Definitions** | **Reference** |
| --- | --- | --- | --- |
| Anderson-Barker | QTc = QT +(-0.4231(242-HR)) | Where QT is in msec and HR in bpm | See manuscript |
| Bazett | QTc = QT/RR^1/2 | Where QT and RR are in secs | ‑(Bazett, 1920) |
| Fridericia | QTc = QT/(RR^1/3) | Where QT and RR are in secs | (Fridericia, 1920) |
| Framingham | QTc = QT + 0.154(1-RR) | Where QT and RR are in secs | (Sagie *et al.*, 1992) |
| Van de Water | QTc = QT-0.087(RR-1000) | Where QT and RR are in msecs | (Van de Water *et al.*, 1989) |
| Benatar* | QTc = QT + α(1-RR) | Where QT and RR are in secs and α is the slope of the QT-RR regression line | (Benatar & Feenstra, 2015) |
| Hodges | QTC = QT+1.75(HR-60) | Where QT is in msec and HR in bpm | (Hodges *et al.*, 1983) |
| Wernicke | QTc = QT /(RR^0.38) | Where QT and RR are in secs | (Wernicke *et al.*, 2005) |

***Note:*** **Benatar and Feenstra (2015) describe an age-specific equation in which the slope of the QT-RR regression line (α) varies with age. In the analysis shown here we have estimated α to be 0.2251 for our cohort of guinea pigs (see Figure 5C).*

**Table S1**: Equations used for the estimation of the rate-corrected QT interval of the ECG (QTc) based on changes in either Heart Rate (HR) or RR interval (RR).

The QTc correction for each of the equations shown in Table 1 are plotted as a function of Heart Rate (Figure S1 and S2) and as a function of RR interval (Figures S3 and S4).

***Figure S2:*** *Corrected QT intervals (QTc) estimated from the various equations shown in Table 1, originally designed for use in adults, plotted as a function of Heart Rate (bpm)*. Slope = the slope of the linear regression line, r2 = ‘goodness of fit’, F is the variance and P the probability that the two variables are related. The ideal characteristics this line are: slope =0, r^2^ =0 and F =0, and P >0.05.

***Figure S3:*** *QTc data calculated using the Benatar and Wernicke equations (Table 1) plotted as a function of Heart Rate (bpm)*.  *These equations were originally devised for use in children.* The regression line data are as described in Figure S2.

***Figure S4:*** *Corrected QT intervals (QTc) estimated from the various equations shown in Table 1, originally designed for use in adults, plotted as a function of RR interval (msec).* The regression line data are as described in Figure S2.

***Figure S5:*** *QTc data calculated using the Benatar and Wernicke equations (Table 1) plotted as a function of RR interval (msec)*.  *These equations were originally devised for use in children.* The regression line data are as described in Figure S2.
